# Potential Implications of miRNAs in the Pathogenesis, Diagnosis, and Therapeutics of Alzheimer’s Disease

**DOI:** 10.3390/ijms242216259

**Published:** 2023-11-13

**Authors:** Long Wang, Xindong Shui, Yuelin Diao, Duoting Chen, Ying Zhou, Tae Ho Lee

**Affiliations:** Fujian Key Laboratory of Translational Research in Cancer and Neurodegenerative Diseases, School of Basic Medical Sciences, Fujian Medical University, Fuzhou 350122, China; wanglong@fjmu.edu.cn (L.W.);

**Keywords:** Alzheimer’s disease, microRNA, tau, amyloid precursor protein, beta-amyloid, APOE, neuroinflammation, diagnosis, therapy

## Abstract

Alzheimer’s disease (AD) is a complex multifactorial disorder that poses a substantial burden on patients, caregivers, and society. Considering the increased aging population and life expectancy, the incidence of AD will continue to rise in the following decades. However, the molecular pathogenesis of AD remains controversial, superior blood-based biomarker candidates for early diagnosis are still lacking, and effective therapeutics to halt or slow disease progression are urgently needed. As powerful genetic regulators, microRNAs (miRNAs) are receiving increasing attention due to their implications in the initiation, development, and theranostics of various diseases, including AD. In this review, we summarize miRNAs that directly target microtubule-associated protein tau (MAPT), amyloid precursor protein (APP), and β-site APP-cleaving enzyme 1 (BACE1) transcripts and regulate the alternative splicing of tau and APP. We also discuss related kinases, such as glycogen synthase kinase (GSK)-3β, cyclin-dependent kinase 5 (CDK5), and death-associated protein kinase 1 (DAPK1), as well as apolipoprotein E, that are directly targeted by miRNAs to control tau phosphorylation and amyloidogenic APP processing leading to Aβ pathologies. Moreover, there is evidence of miRNA-mediated modulation of inflammation. Furthermore, circulating miRNAs in the serum or plasma of AD patients as noninvasive biomarkers with diagnostic potential are reviewed. In addition, miRNA-based therapeutics optimized with nanocarriers or exosomes as potential options for AD treatment are discussed.

## 1. Introduction

Characterized by a time-dependent decline in the function of organisms, aging is a weakening of the body’s defense and repair functions [1,2]. Owing to longer life expectancies in our modern society, a rapidly increasing aging human population has resulted in an elevated global burden of late-life diseases [3]. Notably, aging is associated with increased susceptibility to infections, tumors, including malignant tumors, and neurodegenerative diseases such as Alzheimer’s disease (AD) [4,5]. For individuals above 65 years of age, the odds of developing AD double every five years, and one out of three people aged 85 or older develop AD [6]. AD is the main cause of dementia, and its prevalence is projected to triple by 2050 according to a prevalence estimate of dementia from a study reporting it, reaching approximately 50 million worldwide in 2018 [7]. Thus, AD has emerged as a serious global public health threat as a result of an ever-increasing aging population.

AD is a subtle yet devastating age-related progressive neurodegenerative disorder defined by numerous cognitive or behavioral symptoms [7,8]. Currently, there are four hypotheses for the pathogenesis of sporadic or late-onset AD, including the amyloid cascade, and the inflammatory, vascular, and infectious factors [9]. The cardinal neuropathological hallmarks of AD are aggregation of neurofibrillary tangles (NFTs) due to hyperphosphorylated tau and the accumulation of beta-amyloid (Aβ) processed from amyloid precursor protein (APP) [10,11]. These two features have been recognized for more than one hundred years and they remain indispensable for AD diagnosis today [12]. Unfortunately, the pathogenesis of AD is still unknown. Moreover, early and accurate diagnosis of AD remains an enormous challenge. Furthermore, current disease-modifying therapeutics have been largely unsuccessful. Therefore, thoroughly understanding the molecular changes underlying the pathogenesis of AD may reveal potential targets for diagnosis and therapeutics.

The discovery of microRNAs (miRNAs) has revolutionized our comprehension of gene regulation as they have important target recognition and regulatory functions [13,14]. In human cells, at least 2300 mature miRNAs have been experimentally validated [15]. These miRNAs have been estimated to modulate approximately half of all protein-coding genes via posttranscriptional regulation [16]. The expression profiling of miRNAs has been found to be time- and tissue-dependent [17,18]. Dysregulation of miRNAs is regarded as a reflection of the state of cells in different tissues and a causal factor in various disorders [19,20], suggesting potential implications in the pathogenesis and theranostics of human diseases.

Up to 70% of all miRNAs are expressed in the human nervous system [21]. MiRNAs also play critical regulatory roles in the accumulation of toxic proteins that affect neuronal survival [22]. Importantly, altered expression of several miRNAs has been found in the early stages of AD, around two decades before the onset of clinical symptoms [23]. These findings suggest that miRNAs possess diagnostic and therapeutic value. In this review, we summarize the regulatory roles of miRNAs in tau pathologies and amyloidogenic APP processing resulting in Aβ pathologies in AD. Moreover, we explored the capacity of miRNAs as noninvasive biomarkers for AD diagnosis. Furthermore, miRNA-based therapeutics have been evaluated for potential AD treatment.

## 2. MiRNA-Mediated Regulation of Tau Pathologies in AD

### 2.1. Tau Pathologies in AD

In 1975, a protein named tau was found to be an essential regulator of microtubule assembly [24]. In humans, tau is encoded by microtubule-associated protein tau (MAPT) located on chromosome 17q21, generating six molecular isoforms due to alternative splicing [25]. Tau protein is expressed predominantly in neurons and is vital for the stabilization of the neuronal cytoskeleton [26]. Notably, tau abnormalities have been proven to result in various neurological diseases collectively known as tauopathies, including AD [27].

Among a series of posttranslational modifications such as methylation, acetylation, phosphorylation, and N-glycosylation, phosphorylation strongly correlates with pathological tau conditions [28]. Under normal physiological conditions, the phosphorylation of tau is developmentally modulated and promotes microtubule assembly [29,30]. However, in certain pathological situations, a substantial rise in the phosphorylation of tau has been observed, resulting in aberrant hyperphosphorylated tau [30]. Aberrant tau phosphorylation is regulated by protein kinases and phosphatases [26].

In AD, the abnormal tau phosphorylation not only breaks down neuronal microtubules but also prevents normal tau functioning by facilitating interactions with normal tau [31]. Notably, it is hyperphosphorylated tau instead of normal tau that form a component of NFTs called paired helical filaments (PHFs), thereby contributing to the generation of NFTs [32,33,34]. The resulting aggregation of NFTs correlates with the severity of the cognitive decline [35,36]. Interestingly, tau pathologies have recently been proposed to be essential initiating factors in the sporadic form of AD [37].

### 2.2. MiRNAs Regulate MAPT Expression

#### 2.2.1. MiRNAs Directly Modulate MAPT Transcript Levels

**Figure 1:**
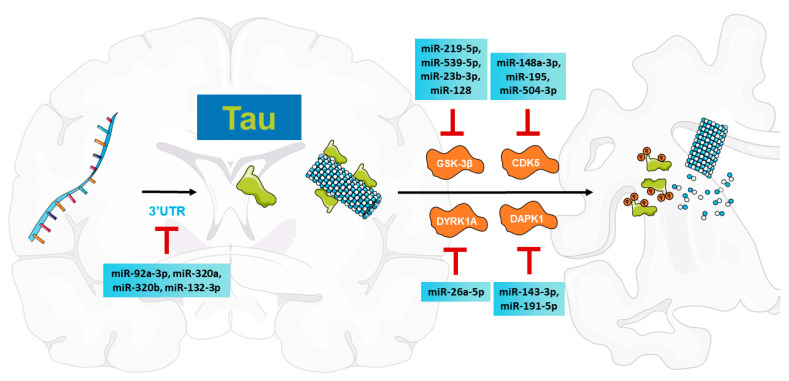
A summary of miRNAs directly targeting MAPT transcripts and related kinases, including GSK-3β, CDK5, DYRK1A, and DAPK1.

Recently, through capture technology, miR-92a-3p, miR-320a, and miR-320b were found to directly bind to MAPT mRNA and inhibit tau protein expression in a human neuroblastoma cell line [38]. Overexpression of these miRNAs leads to a significant decrease in tau levels, while miRNA inhibitors dramatically upregulate the protein expression of tau [38]. Interestingly, the authors further identified that plasma miR-92a-3p levels were reduced in AD individuals when compared with healthy participants, which is consistent with several previous publications [39,40] but contradictory with others [41]. Downregulation of miR-320a has been shown in the serum [39] and cerebrospinal fluid (CSF) [42] of patients with AD versus controls, although contrary data exist [43]. Moreover, miR-132-3p, which is frequently downregulated in AD and other tauopathies, directly targets MAPT mRNA to inhibit its expression, and its deletion in AD model mice leads to tau aggregation [44]. In addition, miR-34c-5p [45], miR-186 [46], and miR-27a [47] can bind to the 3′UTR of MAPT in human cell lines or a rat model; however, these interactions have not been observed in AD. These miRNAs directly target the mRNA of tau, participating in the development of tau pathologies in AD (Figure 1).

#### 2.2.2. MiRNAs Regulate Tau Alternative Splicing

In adult human brains, alternative splicing of exons 2, 3, and 10 of MAPT pre-mRNA generates six tau isoforms [25]. The exclusion or inclusion of exon 10, encoding the second microtubule-binding repeat of tau, results in tau isoforms containing either three (3R) or four (4R) microtubule-binding domain repeats [48]. Approximately equal 3R-tau and 4R-tau are physiologically expressed in adults, but tau exon 10 splicing becomes dysregulated in several tauopathies and alters the ratios between 4R-tau and 3R-tau isoforms, contributing to neurodegeneration and dementia [49]. Several miRNAs, such as miR-124, miR-9, miR-132, and miR-137, have been shown to be involved in the aberrant splicing of tau exon 10 and the modulation of 4R:3R-tau ratios in neuronal cells [50]. Specifically downregulated in the brains of patients with sporadic progressive supranuclear palsy, miR-132 directly targets polypyrimidine tract-binding protein, a neuronal splicing factor that is significantly upregulated in PSP brain regions [50]. Whether and how these miRNAs affect tau splicing in AD remains undetermined. Given that miR-132 [51,52], miR-124 [53,54], miR-9 [55,56], and miR-137 [57,58] are implicated in AD, it would be interesting to further investigate the potential roles of miRNAs in the alternative splicing of tau in AD.

### 2.3. MiRNAs Regulate Kinases That Phosphorylate Tau

The phosphorylation of tau is developmentally modulated, as phosphorylated tau levels are high in fetal brains and decline with age during development [30]. However, in AD brains, tau is aberrantly hyperphosphorylated [25]. Direct pathological events involved in tau hyperphosphorylation include the increase or abnormal activation of kinases [35], such as glycogen synthase kinase (GSK)-3β, cyclin-dependent kinase 5 (CDK5), dual-specificity tyrosine phosphorylation-regulated kinase 1A (DYRK1A), and death-associated protein kinase 1 (DAPK1), which have been found to be directly regulated by miRNAs in tau pathologies (Figure 1).

#### 2.3.1. MiRNAs Modulate GSK-3β to Inhibit Tau Phosphorylation

GSK-3β is a multifunctional serine/threonine kinase enriched in the brain and shows elevated levels with aging [59]. Notably, increased levels of active GSK-3β are observed in AD brains, which are considered an early event before NFT formation [60]. Several miRNAs have been demonstrated to modulate GSK-3β and participate in tau pathologies in AD. MiR-219-5p is downregulated in AD brains and inhibits tau phosphorylation at Ser198, Ser199, Ser201, and Ser422 via directly targeting GSK-3β in a human neuroblastoma cell line [61]. Moreover, in the CSF of AD patients, miR-539-5p levels are dramatically decreased compared with those in the CSF of healthy controls, showing a negative correlation with GSK-3β expression [62]. Upregulation of miR-539-5p through injection in AD model mice downregulates tau phosphorylation at Ser396 and Ser404 and improves memory ability [62]. Furthermore, in the plasma of AD patients, miR-23b-3p is remarkably downregulated compared to that in the plasma of healthy age-matched individuals [63]. MiR-23b-3p exhibits neuroprotection by inhibiting tau phosphorylation, alleviating AD-like symptoms in AD model mice, and interrupting GSK-3β-dependent tau phosphorylation at Ser396 and Ser404 in vivo [63]. In addition, the brain-specific miRNA miR-128 inhibits tau phosphorylation at Ser396, Ser404, and Thr217 by directly suppressing GSK-3β, and an increase in miR-128 levels in the hippocampus improves spatial learning and memory in 5×FAD mice [64].

#### 2.3.2. MiRNAs Are Involved in CDK5 Regulation to Reduce Tau-Related Pathologies

CDK5 is an essential serine/threonine protein kinase and a unique member of the cyclin-dependent kinase family as the activity of CDK5 is restrictedly regulated by the neuronal-specific and membrane-localized activators p35 and p39 (or their respective truncated forms p25 and p29) instead of binding to cyclins in the central nervous system (CNS) [65,66]. In AD brain tissues, an increase in CDK5 immunoreactivity has been found in neurons bearing early-stage NFTs [67]. Increased activity of CDK5 contributes to the accumulation of aggregated tau and promotes neurofibrillary pathology development [68]. In the serum of AD patients, miR-148a-3p expression levels are lower [69]. MiR-148a-3p decreases tau hyperphosphorylation at Ser202/Thr205, Ser199, Ser396, and Ser404 by targeting cyclin-dependent kinase 5 regulatory subunit 1 (CDK5R1) mRNA, which encodes the p35 protein, and intracerebroventricular injections of miR-148a-3p ameliorate cognitive deficits in AD model mice [69]. Moreover, miR-195 overexpression prevents CDK5/p35 activity and tau hyperphosphorylation at Thr231, Ser262, and Ser422 in the hippocampus of model rats by targeting CDK5R1 [70]. Recently, our group found that miR-504-3p binds to the 3′UTR of p39 and downregulates p39 protein expression by modulating its mRNA level [71]. We further demonstrated that miR-504-3p attenuates tau hyperphosphorylation at CDK5-dependent phosphorylation sites, including Thr213 and Ser396, related to AD by targeting p39 [71].

#### 2.3.3. MiRNAs Regulate DYRK1A to Suppress Tau Phosphorylation

DYRK1A is a serine/threonine kinase encoded on human chromosome 21 that plays an important role in early-onset neurodegeneration [72]. In the hippocampus of AD patients, DYRK1A mRNA levels are dramatically increased compared with those in the hippocampus of healthy controls [73]. DYRK1A is involved in the hyperphosphorylation of tau and its extra copy may lead to the early onset of AD [74]. MiR-26a-5p expression is downregulated in the brain tissues of AD model mice and negatively modulates DYRK1A by targeting its 3′UTR [75]. Overexpression of miR-26a-5p inhibits tau phosphorylation at Thr212, Ser202, and Ser404, and alleviates AD-like symptoms in mice [75].

#### 2.3.4. MiRNAs Target DAPK1 to Attenuate Tau Pathologies

DAPK1 is a calcium/calmodulin-regulated serine/threonine kinase that plays essential roles in various types of cancers and neurodegenerative disorders including AD [76,77]. Genetic variations in DAPK1 have been found to show a significant association with late-onset AD [78,79,80,81]. Notably, DAPK1 is highly overexpressed in the hippocampal tissues of AD individuals compared with age-matched controls [82,83,84]. Mechanically, DAPK1 can phosphorylate and inhibit the activity of Pin1, a peptidyl-prolyl cis/trans isomerase that converts cis to trans p-tau to prevent a group of tau pathologies, including AD [8,85,86,87]. Recently, our group found that miR-143-3p inhibits aberrant tau phosphorylation at Thr231, Ser262, and Ser396 and promotes microtubule assembly via directly targeting DAPK1 in AD [88]. Moreover, miR-191-5p was also observed to suppress tau phosphorylation at Thr231, Ser262, and Ser396 and promote neurite outgrowth [89].

## 3. MiRNA-Mediated Modulation of Aβ Pathologies in AD

### 3.1. Amyloidogenic APP Processing in AD

Since the 1990s, the amyloid cascade hypothesis has dominated research and clinical trials of AD [90,91]. Accumulation of Aβ in the brain is hypothesized to drive AD pathogenesis and cause neurodegeneration [92]. Aβ is an aggregation-prone peptide consisting of 36 to 43 amino acids originating from APP proteolysis via the amyloidogenic pathway [6]. The human APP gene is mapped to chromosome 21 and encodes a type I transmembrane protein after the alternative splicing of 18 exons, primarily generating three isoforms, APP695, APP751, and APP770 [93,94,95]. Genomic duplications in the APP locus have been observed to cause early-onset AD [96,97].

APP is metabolized via two distinct proteolytic processes, the nonamyloidogenic pathway and the amyloidogenic pathway [98]. In the nonamyloidogenic processing pathway, APP is first cleaved by α-secretase within the Aβ sequence and further cleaved by γ-secretase, thus precluding intact Aβ generation [99]. However, in the amyloidogenic processing pathway, APP is cleaved by β-secretase (β-site APP-cleaving enzyme 1, BACE1) and subsequently cleaved by γ-secretase, inducing Aβ pathologies [99]. An upregulation in APP expression has also been found in AD brains [100,101]. The aberrant deposition of Aβ is associated with neurotoxicity and has emerged as a neuropathological hallmark of AD [92].

As a single transmembrane-spanning protein, APP possesses a variety of phosphorylation sites in both the short cytoplasmic domain and the long extracellular domain [102]. Within the cytoplasmic domain of APP, there are eight potential phosphorylation sites, seven of which are observed to be phosphorylated in AD brains [102,103]. Phosphorylation of APP is capable of modulating its processing and transport, exerting key regulatory roles in Aβ generation [104,105]. Thus, abnormal phosphorylation of APP and related kinases involved in amyloidogenic APP processing are critical for the development of Aβ pathologies.

### 3.2. MiRNAs Regulate Amyloidogenic APP Processing

#### 3.2.1. MiRNAs Directly Modulate APP mRNA Expression

**Figure 2:**
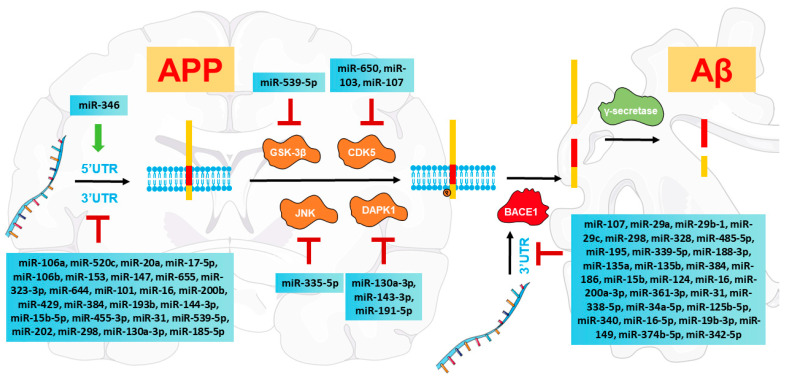
A summary of miRNAs directly targeting APP transcripts, BACE1 transcripts, and related kinases, including GSK-3β, CDK5, JNK, and DAPK1.

In attempts to address whether APP levels can be posttranscriptionally regulated, miR-106a and miR-520c were initially found to target the 3′UTR of APP mRNA and repress its protein expression in human cells [106]. MiR-20a, miR-17-5p, and miR-106b, as members of the same miRNA family, directly regulate APP expression in both human and mouse neuronal cells, while miR-106b is significantly downregulated in sporadic AD patients [107]. MiR-153 negatively regulates APP expression in human neuroblastoma cells, mouse models [108], and primary human fetal brain cultures [109]. MiR-153 levels are dramatically decreased in a subset of advanced AD postmortem brain specimens (Braak stage III-VI) compared with early-stage specimens (control, Braak stage I/II) [109]. In addition to miR-20a, miR-17, and miR-153, miRNAs miR-147, miR-655, miR-323-3p, and miR-644 can also regulate APP expression [110]. MiR-101 modulates APP levels in rat hippocampal neurons [111] and multiple human cell types [112]. MiR-16 directly inhibits APP expression in model mice [113,114] and rat hippocampal neurons [115]. MiR-200b and miR-429 downregulate APP mRNA and protein expression in primary mouse hippocampal neurons and human neuroblastoma cells [116]. MiR-200b levels are lower in the serum of patients with Alzheimer-type dementia than in healthy controls, and Aβ42 can downregulate miR-200b expression, leading to a vicious cycle resulting in the continuous accumulation of Aβ42 [116]. MiR-384 suppresses APP mRNA and protein levels, and APP levels are lower in the CSF and serum of patients with Alzheimer-type dementia [117]. MiR-193b inhibits APP mRNA and protein levels, and exosomal miR-193b levels are reduced in the CSF and blood of AD patients [118]. MiR-144-3p is a negative regulator of APP and inhibits its protein expression in human cells [119]. MiR-15b-5p has been shown to decrease APP mRNA and protein levels in a human AD cell model [120]. MiR-455-3p can decrease APP levels in a mouse neuroblastoma cell line [121]. MiR-31, previously found to be downregulated in AD patients, can reduce APP mRNA levels in human cells and an AD mouse model [122]. MiR-539-5p has also been observed to downregulate APP expression in an AD mouse model [62]. MiR-202 downregulates APP expression, and miR-202 levels are dramatically reduced in the serum of AD patients [123]. MiR-298 is a repressor of APP and downregulates APP expression in a primary human cell culture model [124]. MiR-130a-3p can downregulate the expression of APP in the primary hippocampal neurons of AD model mice [125]. MiR-185-5p can decrease APP transcript levels, and serum exosomal miR-185-5p levels are markedly reduced in the AD group versus the corresponding control groups [126]. All the above-mentioned miRNAs directly target the 3′UTR of APP mRNA to regulate its levels, playing vital roles in the development of Aβ pathologies (Figure 2). Although miR-373-3p can also target APP mRNA and inhibit its protein expression [127], the role of this interaction in AD pathogenesis has not been confirmed. Interestingly, APP 3’UTR polymorphisms located in miRNA target sites have been observed to influence the risk of AD occurrence [110,128]. In addition, miR-346 can target the 5′UTR of APP mRNA to promote APP translation and Aβ generation [129].

#### 3.2.2. MiRNAs Regulate the Alternative Splicing of APP

Apart from the direct regulation of APP by targeting the 5′UTR and 3′UTR in AD, miRNAs also participate in the alternative splicing of APP mRNA. APP695, APP751, and APP770 are the three primary isoforms generated after alternative splicing, and the APP695 isoform is predominantly expressed in neurons [95]. In AD brains, APP695 is generally downregulated, whereas APP751 and APP770 are upregulated [130]. Notably, in postmitotic neurons from the cortex of Dicer conditional knockout mice, the lack of miRNAs results in APP exon 7 and 8 inclusion [131]. MiR-124 is involved in this abnormal APP splicing, and miR-124 levels are decreased in AD brains [131].

#### 3.2.3. MiRNAs Directly Regulate BACE1 Transcripts

APP is first cleaved by BACE1, which is the enzyme that initiates Aβ generation, and BACE1 cleavage of APP is the rate-limiting step for Aβ formation in the brain [132,133]. Initially, BACE1 was cloned and characterized in 1999, and its expression levels and activity have been found to be increased in the brains and body fluids of AD individuals [133]. To investigate the potential regulators contributing to BACE1 posttranscriptional regulation, miR-107 was found to be downregulated early in AD by miRNA expression microarrays, and miR-107 targeting of the 3′UTR of BACE1 mRNA was biochemically validated [134]. Moreover, miR-29a and miR-29b-1 can downregulate BACE1 expression in human neuroblastoma cells, and these miRNAs were found to be downregulated in a cohort of AD patients with aberrantly high BACE1 levels [55]. Belonging to the miR-29 family, miR-29c can also directly inhibit the expression of BACE1 in human cells and mouse models, and its downregulation correlates with the increase in BACE1 levels in sporadic AD patients [135,136,137]. MiR-298 and miR-328 negatively regulate BACE1 expression in cultured neuronal cells [124,138]. MiR-485-5p decreases in BACE1 protein levels in human cells [139]. MiR-195 downregulates BACE1 expression by suppressing its translation in human and mouse cells [140]. MiR-339-5p decreases BACE1 expression levels in human primary brain cultures, and miR-339-5p is downregulated in AD patient brain specimens [141]. MiR-188-3p can downregulate BACE1 expression, and the levels of miR-188-3p are decreased in AD brains [142]. MiR-135a inhibits BACE1 expression in human neuroblastoma cells and primary mouse hippocampal neurons and its levels are reduced in the serum of AD patients [116]. MiR-135b also decreases the protein expression levels of BACE1, and its levels are reduced in the blood of AD individuals [143]. MiR-384 can downregulate BACE1 expression in human neuroblastoma cells as well [117]. Moreover, miR-186 can downregulate BACE1 expression in mouse neuronal cells [144]. MiR-15b modulates BACE1 expression in human neuroblastoma cells, and its levels are decreased in sporadic AD brain tissues [145,146]. MiR-124 levels are downregulated in sporadic AD brain tissues, and miR-124 can inhibit BACE1 expression in human neuroblastoma cells [147] and AD mouse models [148]. MiR-16 downregulates BACE1 expression in an AD cell model, and its levels are decreased in AD brain tissues [149]. MiR-200a-3p inhibits BACE1 protein expression, and its levels are reduced in the blood plasma of AD individuals [150]. MiR-361-3p regulates the expression of BACE1, and its levels are decreased in AD brains [151]. MiR-31 downregulates BACE1 expression in an AD animal model [122]. MiR-338-5p can downregulate BACE1 expression in the hippocampus of AD model mice and is downregulated in the hippocampus of AD patients [152]. MiR-34a-5p and miR-125b-5p reduce BACE1 expression levels in primary mouse cortical neurons, and the levels of these miRNAs are decreased in the serum samples of AD patients [153]. MiR-340 can downregulate BACE1 expression in human neuroblastoma cells [154]. MiR-16-5p and miR-19b-3p can reduce BACE1 protein levels in human neuroblastoma cells [155]. MiR-149 inhibits BACE1 expression in an AD cell model, and its levels are reduced in the serum of AD individuals [156]. MiR-374b-5p has also been verified to interact with BACE1 [157]. MiR-342-5p downregulates BACE1 expression in mouse cells, and its levels are decreased in the circulating small extracellular vesicles from patients with AD [158]. All of these miRNAs directly bind to the 3′UTR of BACE1 mRNA, suggesting that they potentially play roles in modulating Aβ pathologies (Figure 2).

### 3.3. MiRNAs Regulate Kinases That Phosphorylate APP at Thr668

APP is a phosphoprotein with several phosphorylation sites that can be modulated by numerous kinases [104]. Phosphorylation of APP plays regulatory roles in APP processing and APP transport and is thus critical for the generation of Aβ [105]. Among various potential phosphorylation sites, Thr668 in the cytoplasmic region of APP has attracted considerable attention due to its profound impact on APP metabolism [102,103]. APP phosphorylation at Thr668 is increased in the brain samples of AD patients and promotes amyloidogenic APP processing to produce Aβ [103]. The abnormal phosphorylation of APP at Thr668 has been found to be regulated by kinases, such as GSK-3β, CDK5, c-Jun NH2-terminal kinase (JNK), and DAPK1, which have been reported to be directly modulated by miRNAs (Figure 2).

#### 3.3.1. MiRNAs Are Involved in GSK-3β Regulation

GSK-3β is a kinase that is responsible for the Thr668 phosphorylation of APP [159,160]. The activity of GSK-3β is elevated in AD patients [60]. MiR-539-5p has been confirmed to directly target GSK-3β, decrease GSK-3β expression levels, and inhibit Aβ accumulation in APP/PS1 mice [62]. The expression levels of miR-539-5p and GSK-3β are negatively correlated in the CSF and brain tissues of AD patients [62].

#### 3.3.2. MiRNAs Modulate CDK5 to Reduce Aβ Pathologies

CDK5 is also a well-known protein kinase that phosphorylates APP at Thr668 and plays a vital role in APP proteolytic cleavage [161,162]. Hyperactivity of CDK5 has been observed in human AD brains [67]. MiR-650 can target CDK5, and overexpression of miR-650 reduces CDK5 activity and attenuates AD pathologies, including plaque formation and Aβ production, in APP/PSEN1 mice [163]. MiR-103 and miR-107, both members of the miR-15/107 family, can influence CDK5 expression and activity and reduce APP phosphorylation at Thr668 [164]. A significant inverse correlation of expression also exists between these miRNAs and CDK5R1 in AD hippocampal tissues [164].

#### 3.3.3. MiRNAs Target JNK to Inhibit Aβ Production

JNK also plays a fundamental role in the phosphorylation of APP at Thr668 [165,166]. The phosphorylation of JNK is elevated in the postmortem brain tissues of AD individuals [167]. MiR-335-5p can directly bind to the 3′UTR of JNK3 mRNA, decrease its protein levels, and alleviate Aβ accumulation in a human AD neuronal cell model [168]. The decrease in miR-335-5p levels is negatively correlated with the increased levels of JNK3 in AD brain tissues [168].

#### 3.3.4. MiRNAs Regulate DAPK1 to Alleviate Amyloidogenic APP Processing

DAPK1, without its kinase-deficient mutant, has been shown to promote APP phosphorylation at Thr668, initiate APP amyloidogenic processing, and increase Aβ secretion [83]. MiR-130a-3p can target the 3′UTR of DAPK1 and improve the cognitive function of APP/PS1 mice [169]. Recently, our studies indicated that miR-143-3p decreases APP phosphorylation at Thr668 and reduces Aβ40 and Aβ42 production by directly targeting DAPK1 [88]. Moreover, miR-191-5p was also found to inhibit DAPK1, decrease APP phosphorylation levels, and reduce Aβ secretion [89].

### 3.4. MiRNAs Regulate Apolipoprotein E (APOE)-Mediated Aβ Pathologies

Polymorphism in the APOE gene is the strongest genetic risk factor for late-onset AD [170]. Mounting evidence suggests that genetic variation in the APOE gene increases the risk of AD by driving Aβ pathologies [171]. Intriguingly, by evaluating a sample of female patients aged 55 years or older carrying the ε4 allele of APOE, a median 3-fold decrease in miR-9-5p levels was observed when compared with controls, which may be implicated in accelerating amyloidogenic processing [172]. In the plasma of AD patients, miR-1908 is upregulated and is negatively associated with APOE levels [173]. MiR-1908 can directly target the 3′-UTR of APOE mRNA, modulating APOE-mediated Aβ clearance [173].

## 4. MiRNA-Mediated Modulation of Inflammation in AD

Among patients with AD, upregulated levels of inflammatory markers and AD risk genes in association with innate immune functions have been unveiled, suggesting a prominent role of inflammation in the pathogenesis of AD [174]. The inflammatory process includes the activation of microglia and the production of pro-inflammatory cytokines [175]. Notably, microglial activation has been proven to be a vital factor linking the deleterious effects of Aβ to tau spread [176]. The concomitant presence of Aβ, tau, and microglial activation abnormalities emerges as the strongest predictor of cognitive impairment [176]. Several miRNAs have been found to be strongly associated with inflammation in AD.

### 4.1. MiRNAs Induce Pro-Inflammatory Responses

MiR-155 acts as a central pro-inflammatory mediator of neuroinflammation of the CNS [177]. Expressions of a number of pro-inflammatory cytokines, including interleukin (IL)-1β, IL-6, tumor necrosis factor (TNF)-α, and interferon regulatory factor 3 (IRF3), can be upregulated by miR-155 [178]. MiR-155 is involved in the innate immune response pathways and proamyloidogenic pathways leading to Aβ [179]. In microglia, pro-inflammatory miR-155 overexpression can downregulate fibrillar Aβ_1-42_ catabolism [180].

### 4.2. MiRNAs Promote Anti-Inflammatory Responses

MiR-146 is another key regulator of innate immune responses by targeting several inflammation-related mRNAs [181]. As a negative regulator, miR-146 can bind to the mRNA 3′UTRs of the TNF receptor-associated factor 6 and IL-1 receptor-associated kinase 1, controlling the Toll-like receptor and cytokine signaling [182]. Pro-inflammatory cytokines such as IL-6 and TNF-α are also demonstrated to be negatively modulated by miR-146 [183]. Intriguingly, the interactions between miR-155 and miR-146 may result in microglia activation [183].

## 5. Diagnostic Potential of miRNAs in AD

The occurrence of AD doubles every five years for individuals older than 65 years of age [6]. Currently, few effective disease-modifying drugs can prevent or reverse this devastating disorder [184]. One explanation for numerous failed clinical trials might be that the neuropathological alterations of AD start 10–20 years before detectable clinical onset [184,185]. Hence, it is crucial to identify potential biomarker candidates for early diagnosis and disease progression monitoring. A biological rather than a syndromal definition of AD has been defined by the intravital diagnosis of AD, which may better characterize and understand the disease [186]. Currently, CSF and positron emission tomography (PET) biomarkers, including Aβ, tau, and neurofilament light chain (NfL), are employed for the prediction of dementia onset and progression from mild cognitive impairment (MCI) to AD [187,188]. However, these costly, inconvenient, and invasive strategies limit their use as first-line diagnostic tools.

Notably, blood-based biomarkers have attracted considerable attention since they can offer noninvasive, easily accessible, cost-effective, fast, real-time, and repeatable approaches to provide valuable early diagnostic and prognostic insights into multifarious diseases, including cancers and AD [189]. In AD, blood-based biomarkers hold great potential for both primary care screening and personalized precision medicine [189]. Interestingly, miRNAs in blood are stable, providing invaluable diagnostic, prognostic, and predictive information [190,191]. Circulating miRNAs associated with extracellular vesicles such as exosomes present remarkable stability even at room temperature [192]. Importantly, ten miRNAs deregulated both in the bloodstream and in the brain of Braak stage III AD patients have been reported to have diagnostic value nearly two decades prior to clinical symptom onset, offering attractive early miRNA biomarkers for AD [23].

To determine the potential of miRNAs as early noninvasive markers for the diagnosis of AD, various screening strategies have been carried out. After assessing 654 human miRNAs, a unique circulating 7-miRNA signature was validated to be downregulated in the plasma of AD individuals to distinguish AD patients from normal controls [193]. Employing next-generation sequencing to miRNAs from blood samples, a 12-miRNA signature was observed to separate AD patients from healthy controls [194]. Six previously reported miRNAs dysregulated in AD were further examined, and serum miR-125b alone was able to distinguish AD patients from healthy controls [195]. Moreover, via genome-wide serum miRNA expression analysis, a serum miRNA panel consisting of six miRNAs or miR-342-3p alone was shown to be able to distinguish AD patients from healthy controls [196]. By determining the expression of 84 miRNAs, the levels of miR-125b, miR-23a, and miR-26b in serum were validated to be decreased in AD patients, and serum miR-125 levels can distinguish AD patients from healthy controls [197]. Based on Solexa sequencing analysis, the levels of miR-31, miR-93, miR-143, and miR-146a were confirmed to be reduced in the serum of AD patients compared with healthy controls, and this panel can be used to discriminate AD patients from healthy controls [198]. By using omiRas, a total of 27 miRNAs with differential expression between AD patients and healthy controls were identified, and this panel can separate several AD subgroups from healthy controls [199]. Using next-generation deep sequencing, 16 dysregulated miRNAs in exosomes isolated from serum were selected for predicting AD [200]. Among 20 plasma exosomal miRNAs that presented differential expression in AD resulting from initial screening after deep sequencing, a panel of seven miRNAs was reported to predict AD status [201]. In a parallel whole-blood-based study on the seven miRNAs, a decrease in the levels of miR-9-5p, miR-106a-5p, miR-106b-5p, and miR-107 was found to be correlated with a higher risk of AD [202]. By high-throughput next-generation sequencing, serum miR-501-3p levels were observed to be decreased in AD patients and correlated with Mini-Mental State Examination (MMSE) scores [203]. Among 179 miRNAs assessed in the plasma, six miRNAs were selected to differentiate AD from healthy controls [43]. Among 10 mature miRNAs dysregulated in the plasma of AD patients, miR-34a-5p and miR-545-3p were observed to present diagnostic accuracy distinguishing AD patients from control subjects [204]. Using microarray analysis, miR-455-3p was validated to be increased in the serum of AD patients and was able to distinguish individuals with or without AD [205]. Through next-generation sequencing, a 9-miRNA signature in serum was utilized for detecting AD [206]. By examining miRNAs related to synaptic proteins in the plasma of AD subjects, the levels of miR-92a-3p, miR-181c-5p, and miR-210-3p were found to be increased and were able to distinguish AD patients from healthy controls [41]. Based on miRNA profiling in patient plasma, miR-206 levels were found to be elevated in AD patients and predict cognitive decline [207]. In a machine learning approach, a serum 12-miRNA signature was constructed to discriminate AD patients from healthy controls [208]. Among 853 miRNAs in the blood samples, a panel of six dysregulated exosomal miRNAs was selected to detect preclinical AD [209]. Using capture technology, plasma miR-92a-3p and miR-320a among miRNAs directly binding to MAPT mRNA were validated to discriminate AD patients from healthy controls [38].

The detailed miRNAs described in the above-mentioned screening processes are listed in Table 1. In addition, miR-137, miR-181c, miR-9, miR-29a, and miR-29b in the serum [210], miR-34a and miR-146a in the plasma [211], miR-34c in the plasma [212], miR-210 in the serum [213], miR-223 in the serum [214], miR-29c-3p and miR-19b-3p in the serum [215], miR-135a, miR-193b, and miR-384 in the serum [216], miR-133b in the serum [217], miR-103 and miR-107 in the plasma [218], miR-28-3p in the serum [219], miR-483-5p in the plasma [220], miR-331-3p in the serum [221], miR-128 in the serum [222], and miR-106b in the serum [223] have also been observed to be dysregulated in AD patients compared with healthy controls and serve as potential biomarkers to predict AD. In addition, the miR-132 family (miR-128, miR-132, and miR-874) normalized per miR-491-5p and the miR-134 family (miR-134, miR-323-3p, and miR-382) normalized per miR-370 in the plasma [224,225], miR-107 in the plasma [226], miR-206 in the serum and plasma [207,227], miR-132 in the plasma [228], and miR-1185-2-3p, miR-1909-3p, miR-22-5p, miR-134-3p, and miR-107 in the plasma [229] have been shown to play predictive roles in MCI diagnosis and progression from MCI to AD.

Nevertheless, a series of limitations need to be addressed. Gender differences and variations among different human populations may exist for some circulating miRNAs as peripheral biomarkers in AD [230,231]. A standardized and reliable method or equipment with high specificity and sensitivity is needed for miRNA detection. Further investigations are also needed to validate the correlations of these blood-based biomarkers with established AD biomarkers in a larger cohort of participants.

## 6. Therapeutic Potential of miRNAs in AD

In recent decades, AD has rapidly become one of the most hindering and costly disorders affecting the elderly with a high mortality rate, emerging as a worldwide burden for both patients and caregivers [7]. Unfortunately, effective disease-modifying therapeutics are still lacking [184]. Since advanced age is considered the most influential risk factor for AD, antioxidants, including melatonin and resveratrol, have been regarded as potential candidates for neuroprotection against aging. Recently, we observed that melatonin alleviates tau pathologies by upregulating miR-504-3p expression [71], suggesting that miRNAs play critical roles in AD treatment. Resveratrol can also rescue aberrant expression of miRNAs to exert neuroprotective effects [232]. Currently, the majority of drug development programs are targeting Aβ and tau [7]. Nevertheless, the resulting clinical trials have been disappointing despite the positive therapeutic outcomes in cell and animal models [233]. Thus, more therapeutic approaches are needed to confront this complex multifactorial disorder.

Compared with conventional therapeutic methods that target proteins instead of underlying causes resulting in transient effects, employing nucleic acids as therapeutics may trigger long-lasting or curative effects due to the powerful capacities of gene inhibition, addition, replacement, or editing [234]. Due to their considerable merits of specificity, safety, and suitability for targets that remain undruggable, nucleic acid therapeutics possess the potential to emerge as the third pillar of drug development in addition to small molecule inhibitors and antibodies [235]. As one of the most advanced and efficacious therapeutic options for rare genetic disorders and debilitating diseases, nucleic acid therapeutics can achieve precise targeting with sequence-specific nucleic acid recognition, opening new avenues for CNS disease treatment [236]. Over the last quarter century, a total of 18 nucleic acid therapeutics have received clinical approval for treating various diseases [237]. Notably, among approximately 20,000 human proteins, only approximately 3000 proteins are druggable, and fewer than 700 proteins can be targeted by approved drugs [238,239]. Therefore, nucleic acid therapeutics with abundant candidate targets and relatively simple preparation processes may provide novel insights into targeting previously undruggable proteins to treat diseases that are difficult to cure, such as AD.

Recently, miRNA-based therapeutics that replenish or inhibit miRNA function via delivery of synthetic small RNA molecules have drawn considerable interest and shown promise in preclinical studies with multiple advantages [240]. First, miRNAs are natural molecules that originally exist in human cells and are thus involved in biological processes in vivo [241]. Second, in contrast to conventional therapeutics, miRNAs can directly bind to downstream targets to modulate their expression. Moreover, the size of miRNAs can facilitate the development of reliable and effective delivery systems. Furthermore, miRNAs may simultaneously modulate several genes within one specific pathway, leading to a more robust yet specific regulation [241]. As synthetic double-stranded oligonucleotides, miRNA mimics play roles in restoring lost miRNA function among different types of diseases [240,241]. To date, two miRNA mimics, MRX34 (miR-34 mimics) and MesomiR-1 (miR-16 mimics), have been tested in clinical trials for cancer treatment [241]. The potential clinical applications of miRNA-based therapeutics for AD treatment remain to be exploited.

Notably, despite the appealing therapeutic use of miRNAs, numerous challenges need to be overcome, including poor stability, low cell membrane permeability, the existence of the blood-brain barrier (BBB), and the limited targeting of specific tissues [18]. Naked RNA molecules are prone to degradation in circulation, and their negative charges as well as large size make it difficult for them to cross the cell membrane [242]. The presence of the BBB further hinders RNA uptake and delivery into brain tissues [239]. The rapid advancements in nanotechnology provide new possibilities for therapies for brain disorders with promising delivery systems. To address the issue of BBB penetration, we previously developed a BBB-permeable nanocapsule with 2-methacryloyloxyethyl phosphorylcholine (MPC) on the surface [243]. The nanocapsule is able to cross the BBB and release its cargo in the brain [243]. To date, a wide variety of nanocarriers have been fabricated with improved RNA delivery efficiency and minimized toxicity [242]. Both conventional and advanced nanocarriers are being extensively explored for effective drug delivery in the treatment of AD [244,245,246]. As naturally secreted nanosized extracellular vesicles, exosomes have sparked great interest as vehicles for the delivery of drugs due to their unique characteristics, including increased blood stability, reduced cytotoxicity, and limited immunogenicity [247]. Recently, we functionalized exosomes with the peptide angiopep-2, resulting in efficient nanocarriers with enhanced BBB permeability and improved biosafety [248]. In AD, exosomes have been demonstrated to restore cognitive function in animal models, offering an attractive therapeutic tool for treatment [249,250,251]. Thus, well-designed artificial and natural nanocarriers may facilitate therapeutic miRNA delivery in AD.

## 7. Concluding Remarks

In conclusion, miRNAs can directly regulate MAPT transcripts, the alternative splicing of tau, and related kinases to modulate tau, playing key roles in tau pathologies in AD. Moreover, miRNAs can directly modulate APP transcripts, the alternative splicing of APP, BACE1 transcripts, and related kinases to control amyloidogenic APP processing, exerting regulatory effects on Aβ pathologies in AD. Circulating miRNAs in blood have emerged as potential noninvasive biomarkers for AD diagnosis. The appealing potential of miRNA-based therapeutics holds promise, exploiting advanced delivery systems such as nanocarriers and exosomes. While miRNAs play a crucial role in regulating gene expression in a sequence-specific manner, the dual nature of their ability to modulate multiple targets poses a complex scenario. The question arises of whether miRNAs function as precise and specific regulators or if their impact is more potent within a particular pathway compared to a combination of siRNAs. Further investigation is required to clarify this aspect. With more insights into the role of miRNAs in AD pathogenesis and growing interest in developing novel technologies, miRNAs are expected to have diagnostic and therapeutic applications in the coming decades.

## Figures and Tables

**Table 1:** Identification of miRNAs as potential blood-based biomarkers for AD diagnosis through screening processes.

miRNA Profile	Sample	Project Description	References
miR-let-7d-5p, miR-let-7g-5p, miR-15b-5p, miR-142-3p, miR-191-5p, miR-301a-3p, and miR-545-3p	plasma	These 7 signature miRNAs are downregulated in the plasma of AD patients and can discriminate AD individuals from healthy controls with >95% accuracy (AUC of 0.953).	[193]
miR-112, miR-161, miR-let-7d-3p, miR-5010-3p, miR-26a-5p, miR-1285-5p, and miR-151a-3p, miR-103a-3p, miR-107, miR-532-5p, miR-26b-5p, and miR-let-7f-5p	blood	These 12 signature miRNAs can discriminate AD patients from healthy controls with an accuracy of 93%, a specificity of 95%, and a sensitivity of 92%.	[194]
miR-125b	serum	The level of this miRNA is downregulated in the serum of AD patients, distinguishing AD individuals from healthy controls with a specificity up to 68.3% and a sensitivity of 80.8% and is correlated with the MMSE in AD patients.	[195]
miR-98-5p, miR-885-5p, miR-483-3p, miR-342-3p, miR-191-5p, and miR-let-7d-5p	serum	These miRNAs are downregulated in AD patients, while miR-342-3p has the best sensitivity (81.5%) and specificity (70.1%) and is correlated to MMSE score.	[196]
miR-125b, miR-23a, and miR-26b	serum	The levels of these miRNAs are decreased in the serum of AD patients, and serum miR-125 levels can distinguish AD individuals from healthy controls with an accuracy of 82%.	[197]
miR-31, miR-93, miR-143, and miR-146a	serum	The levels of these miRNAs are decreased in the serum of AD patients, and this panel can be used to distinguish AD individuals from healthy controls with an AUC more than 0.7.	[198]
miR-26b-3p, miR-28-3p, miR-30c-5p, miR-30d-5p, miR-148b-5p, miR-151a-3p, miR-186-5p, miR-425-5p, miR-550a-5p, miR-1468, miR-4781-3p, miR-5001-3p, miR-6513-3p, miR-let-7a-5p, miR-let-7e-5p, miR-let-7f-5p, miR-let-7g-5p, miR-15a-5p, miR-17-3p, miR-29b-3p, miR-98-5p, miR-144-5p, miR-148a-3p, miR-502-3p, miR-660-5p, miR-1294, and miR-3200-3p	blood	These miRNAs are differentially expressed between AD and control groups. The entire 27 miRNA panel can distinguish several AD subgroups from controls with an accuracy of 0.801, a sensitivity of 70.8%, and a specificity of 81.8%.	[199]
miR-1306-5p, miR-342-3p, miR-18b-5p, miR-20a-5p, miR-30e-5p, miR-582-5p, miR-106a-5p, miR-361-5p, miR-143-3p, miR-424-5p, miR-93-5p, miR-106b-5p, miR-101-3p, miR-15b-3p, miR-335-5p, and miR-15a-5p	serum	These miRNAs can distinguish AD participants from healthy controls with a sensitivity of 87% and a specificity of 77%.	[200]
miR-185-5p, miR-342-3p, miR-141-3p, miR-342-5p, miR-23b-3p, miR-338-3p, and miR-3613-3p	plasma	These miRNAs can predict AD status with an accuracy of 83–89% in a machine learning model.	[201]
miR-9-5p, miR-106a-5p, miR-106b-5p, and miR-107	blood	These miRNAs are associated with the risk of AD, and miR-106a-5p as a predictor variable shows a specificity of 93% and a sensitivity of 68%.	[202]
miR-501-3p	serum	The level of this miRNA is decreased in the serum of AD patients and shows a sensitivity of 53% and a specificity of 100% with an AUC of 0.82. Its lower levels are associated with lower MMSE scores.	[203]
miR-483-5p, miR-486-5p, miR-30a-5p, miR-200a-3p, miR-502-3p, and miR-142-3p	plasma	These miRNAs can distinguish AD patients from healthy controls with specificities from 0.78 to 1 and sensitivities from 0.75 to 1.	[43]
miR-34a-5p and miR-545-3p	plasma	These miRNAs are downregulated in AD samples and show suitable diagnostic accuracy to distinguish AD patients from healthy controls. The AUC for miR-34a-5p was 0.77 with a sensitivity of 76.19% and a specificity of 71.43%. The AUC for miR-545-3p was 0.75 with a sensitivity of 94.12% and a specificity of 76.01%.	[204]
miR-455-3p	serum	The level of this miRNA is increased in the serum of AD individuals and can distinguish AD patients from healthy controls with an AUC of 0.79.	[205]
miR-26a-5p, miR-181c-3p, miR-126-5p, miR-22-3p, miR-148b-5p, miR-106b-3p, miR-6119-5p, miR-1246, and miR-660-5p	serum	These miRNAs can distinguish AD patients from controls with the AUC between 70.0% and 85.3%. Among the 9 miRNAs, miR-22-3p has the best sensitivity of 81.8% and a specificity of 70.9%.	[206]
miR-92a-3p, miR-181c-5p, and miR-210-3p	plasma	The levels of these miRNAs are increased in the plasma of AD patients and can distinguish AD individuals from healthy controls with an AUC value of 0.855, a sensitivity of 92.86%, and a specificity of 71.43%.	[41]
miR-206	plasma	The level of this miRNA is increased in the plasma of AD patients and can predict cognitive decline using the MMSE test with a sensitivity of 87.50% and a specificity of 77.78%.	[207]
miR-346, miR-345-5p, miR-122-3p, miR-208b-3p, miR-1291, miR-640, miR-499a-5p, miR-650, miR-1285-3p, miR-1299, miR-1267, and miR-206	serum	The levels of these miRNAs in the serum can distinguish AD patients from healthy controls with an accuracy of 76.0%, a sensitivity of 90.0%, and a specificity of 66.7%.	[208]
miR-29c-5p, miR-143-3p, miR-335-5p, miR-485-5p, miR-138-5p, and miR-342-3p	blood	These miRNAs can predict preclinical AD at the asymptomatic stage 5 to 7 years prior to cognitive impairment onset with an AUC of 0.852.	[209]
miR-92a-3p and miR-320a	plasma	These miRNAs can directly bind to the MAPT mRNA and distinguish AD patients from healthy controls. MiR-92a-3p can distinguish AD individuals from healthy controls with an AUC of 0.76 and a sensitivity of 63%. The miR-320a can distinguish AD subjects from controls with an AUC of 0.73 and a sensitivity of 84%.	[38]

Abbreviations: AUC: area under the curve; MMSE: Mini-Mental State Examination.

## Data Availability

No new data were created or analyzed in this study. Data sharing is not applicable to this article.
